# Durvalumab as consolidation therapy in patients who received chemoradiotherapy for unresectable stage III NSCLC: Real-world data from an expanded access program in Brazil (LACOG 0120)

**DOI:** 10.36416/1806-3756/e20240228

**Published:** 2024-12-17

**Authors:** Mauro Zukin, Victor Gondim, Andrea Kazumi Shimada, Ellias Magalhães e Abreu Lima, Clarissa Mathias, Williams Fernandes Barra, William Nassib William, Mônica Padoan, Yuri Bittencourt, Rosely Yamamura, Carlos Eduardo Baston Silva, Letícia de Jesus Rossato, Caio de Almeida Monteiro, Rafaela Gomes de Jesus, Gustavo Gössling, Ana Caroline Zimmer Gelatti

**Affiliations:** 1. Oncologia D’Or e Instituto D’Or, Rio de Janeiro (RJ), Brasil.; 2. Instituto Américas, Rio de Janeiro (RJ), Brasil.; 3. Hospital Sírio-Libanês, São Paulo (SP), Brasil.; 4. Instituto Mario Penna, Belo Horizonte (MG), Brasil.; 5. Oncoclínicas, Salvador (BA), Brasil.; 6. Hospital Santa Izabel, Salvador (BA), Brasil.; 7. Universidade Federal do Pará, Núcleo de Pesquisa em Oncologia, Belém (PA), Brasil.; 8. BP - A Beneficência Portuguesa de São Paulo, São Paulo (SP), Brasil.; 9. Grupo Oncoclínicas, São Paulo (SP), Brasil.; 10. Hospital de Amor, Barretos (SP), Brasil.; 11. Hospital São Lucas, Pontifícia Universidade Católica do Rio Grande do Sul, Porto Alegre (RS), Brasil.; 12. Latin American Cooperative Oncology Group (LACOG), Porto Alegre (RS), Brasil.; 13. Centro de Pesquisa em Oncologia, Hospital São Lucas, Pontifícia Universidade Católica do Rio Grande do Sul, Porto Alegre (RS), Brasil.

**Keywords:** Non-Small-Cell Lung Cancer, Durvalumab, Consolidation Therapy, Real-World Data, Brazil

## Abstract

**Objective:**

The PACIFIC trial established standard therapy for patients with unresectable stage III NSCLC who did not progress after platinum-based concurrent chemoradiation therapy. However, real-world data, particularly from Latin America, remain limited. The LACOG 0120 study aimed to evaluate the efficacy and safety of consolidation therapy with durvalumab in a real-world setting in Brazil.

**Methods:**

Patients with unresectable stage III NSCLC who received chemoradiotherapy followed by durvalumab consolidation therapy through an expanded access program were evaluated. The primary objective was to assess progression-free survival (PFS). Secondary endpoints included overall survival (OS), treatment compliance, and safety, with a focus on the incidence and severity of immune-mediated adverse events (NCT04948411).

**Results:**

Thirty-one patients from seven centers were evaluated. Median follow-up was 50.3 months (95% CI: 48.6-54.4). Median PFS was 9.9 months (95% CI: 7.3-52.4), with a 36 month-PFS of 34.5% (95% CI: 17.7-52.1). Median OS was 34.9 months (95% CI: 26.0-NR), and the 36 month-OS was 46.3% (95% CI: 25.7-64.6). Durvalumab was administered for a median of 17 cycles (10 to 24), with 45.2% of patients completing the planned therapy. The main reason for discontinuation was disease progression. Treatment-related adverse events of any grade occurred in 12 patients (38.7%), with grade 3 events reported in two (6.5%). Pneumonitis was observed in 4 patients (12.9%) - grade 3 in 1 patient.

**Conclusions:**

PFS was lower in this analysis compared to the PACIFIC trial; however, OS was similar, indicating comparable efficacy in a real-world setting among Brazilian patients with unresectable stage III NSCLC. No new safety concerns were identified.

## INTRODUCTION

Non-small-cell lung cancer (NSCLC) continues to be a major global health concern. In 2020, it was the leading cause of cancer-related deaths worldwide, ranking first among men and second among women.^1^ In Brazil, the National Cancer Institute (INCA) reported 16,009 deaths in men and 12,609 deaths in women in 2020,^2^ along with an estimated 32,560 new cases in 2023. These figures, however, may be underestimated due to significant underdiagnosis and underreporting.^3^ Despite this, both Brazil and the United States have observed a decline in the incidence of lung cancer, although a rise in the proportion of localized disease diagnoses was reported in the US.^3^ ^,^ ^4^ Nevertheless, approximately one-third of patients continue to be diagnosed with locally advanced tumors.^1^

For decades, concomitant platinum-based chemotherapy and radiotherapy was the standard of care for patients with unresectable stage III NSCLC and good performance status.^5^ ^,^ ^6^ ^)^ Despite its curative intent, the median progression-free survival (PFS) was only 8 months, with a 5-year overall survival (OS) of only 15% to 30%. Recently, the phase III PACIFIC trial (NCT02125461) evaluated consolidation therapy with anti-programmed cell death ligand-1 (anti-PD-L1) in patients with unresectable stage III NSCLC who did not experience disease progression after chemoradiotherapy (CRT). The results showed that durvalumab significantly improved PFS, with a median of 16.9 months in the experimental group compared to 5.6 months in the placebo group.^7^ Furthermore, the median OS was 47.5 months in the durvalumab group versus 29.1 months in the placebo arm.^7^ As a result, consolidation therapy with durvalumab for 12 months has become the standard of care for patients with unresectable, locally advanced NSCLC.

In Brazil, the National Health Surveillance Agency (*Agência Nacional de Vigilância Sanitária* [ANVISA]) approved this therapeutic modality for unresectable stage III NSCLC in July 2018. Despite the benefits demonstrated in the long-term follow-up of the PACIFIC trial,^7^ at the time of designing this study, the treatment had not yet been approved in the public healthcare system. Recently, however, the National Commission for the Incorporation of Technologies into the Unified Health System (*Comissão Nacional de Incorporação de Tecnologias no Sistema Único de Saúde* [CONITEC]) approved durvalumab consolidation therapy, though it is not yet widely available. While most patients tolerate immunotherapy well, serious immune-mediated adverse events (imAEs), which may hinder its therapeutic benefits, have been reported. Although these events were generally well managed in clinical trials, further validation in a less selected population is warranted.^7^ ^,^ ^8^ ^)^

The PACIFIC trial, conducted across 235 centers in six countries, demonstrated the benefits of immune checkpoint inhibitors (ICI) in 12-month consolidation therapy with durvalumab. Real-world data have validated these benefits; however, further validation is needed in Latin American populations.^9^ ^-^ ^13^ The LACOG 0120 trial sought to evaluate durvalumab consolidation therapy following CRT in the Brazilian population, which, to the best of our knowledge, still lacks real-world data confirming the benefits of consolidation immunotherapy.

## METHODS

The LACOG 0120 trial (NCT04948411) was a non-interventional, observational, retrospective cohort study based on the PACIFIC trial’s Early Access Program (EAP) in Brazil. Eligibility criteria included patients with stage III NSCLC who were treated concurrently or sequentially with platinum-based chemoradiation therapy, received at least one dose of durvalumab in the EAP, and showed no evidence of disease progression. These patients were the first in Brazil to receive this therapy, both before and shortly after its approval by the national regulatory authority.

The characteristics and treatment patterns of patients with stage III NSCLC were retrospectively collected from their medical records, while disease status and survival data were gathered retrospectively or prospectively until all patients completed a minimum 3-year follow-up. All patients enrolled in the EAP underwent definitive CRT (concurrent or sequential) and subsequently received durvalumab until disease progression or intolerability, with no fixed maximum duration-diverging from the 12-month treatment duration specified in the PACIFIC trial. Furthermore, maintenance therapy could be continued despite disease progression if the investigator believed the patient was still benefiting from the intervention. Unlike the original PACIFIC trial, which required durvalumab therapy to begin within a maximum of 42 days,^8^ Brazilian approval for this consolidation therapy imposed no such restriction. However, the LACOG 0120 investigators set a limit of 3 months for starting treatment to allow time for baseline disease assessment and recovery from adverse events related to definitive therapy, without delaying immunotherapy initiation to the point of losing the immunogenic boost from chemoradiotherapy. The participants’ evaluations also included assessments of disease and medical history, physical examinations, biochemical and hematological assays, and documentation of concomitant medication use.

The primary objective was to describe the PFS of patients treated with durvalumab following definitive chemoradiotherapy for stage III, unresectable NSCLC in Brazil. PFS was defined as the time from the initiation of durvalumab treatment to disease progression or death from any given cause, whichever occurred first. It was measured using the Response Evaluation Criteria in Solid Tumors (RECIST) method, version 1.1.^14^

The secondary objective was to determine overall survival (OS), defined as the time from the start of durvalumab treatment to death from any cause. Safety was also a secondary objective and included the rates of temporary and permanent treatment discontinuation, the incidence and severity of adverse events, the duration of treatment interruptions due to adverse events, reasons for treatment discontinuation, and the concomitant use of corticosteroids, immunosuppressants, endocrine therapies, and antibiotics.

Demographic characteristics such as age, sex, and smoking status, as well as clinical aspects, including disease stage, performance status, NSCLC histology, response to CRT, immunohistochemistry, and molecular testing, were described and evaluated. Additionally, details regarding the strategy and duration of CRT, the number of cycles, the type of chemotherapy used, and RT specifics were also assessed.

To describe the baseline stage, the 8th edition of the Tumor Node Metastasis (TNM) classification from the International Association for the Study of Lung Cancer (IASLC) and the Union for International Cancer Control (UICC) was used.^15^ In addition, treatment patterns at the time of disease progression were analyzed, including the duration and type of therapy. Finally, the exploratory objective was to assess the impact of durvalumab consolidation therapy on clinical outcomes and the role of PD-L1 expression in the response to immunotherapy.

Safety was analyzed through physical examinations, the Eastern Cooperative Oncology Group performance status (ECOG PS),^16^ hematological tests, and assessments of renal and thyroid function, liver injury, and metabolic markers. Tumor evaluations were guided by RECIST version 1.1. However, as these evaluations were not mandatory by the study protocol and were performed according to the local standard of care, data on objective responses, disease stability, progression, and death were collected when available from the medical records. The objective response rate (ORR) was defined as the proportion of patients achieving a partial or complete response, as assessed by RECIST version 1.1.

The investigators maintained accurate and complete data records by using electronic case report forms and ensuring the application of informed consent forms, approvals, and notifications where applicable. Data were collected from patient charts and recorded in REDCap, an electronic data capture system, which was supplemented by a series of programmed data-quality checks to automatically detect and prevent the entry of out-of-range or anomalous data. 

The trial protocol was approved by an institutional review board and conducted in accordance with Brazilian regulations and Good Clinical Practice guidelines. It was also approved by the Institutional Ethics Committee.

### Statistical Analyses

The patient sample in the present study was based on the number of patients enrolled in the durvalumab EAP study; no hypothesis testing or power analysis was conducted. Patients who received at least one dose of durvalumab were included in the intention-to-treat population.

Descriptive statistics were used to summarize demographic, clinical, and treatment characteristics, as well as to assess safety. The ORR was calculated with the corresponding 95% two-sided confidence interval (95% CI) using standard methods based on binomial distribution. Estimates of median PFS and OS, along with their respective 95% CIs, were determined using the Kaplan-Meier method.

In order to describe the treatment effect in detail, we evaluated the duration of treatment, the time from the end of CRT to durvalumab initiation, treatment interruptions due to adverse events, reasons for treatment discontinuation, and the need for concomitant use of corticosteroids, immunosuppressants, endocrine therapies, or antibiotics.

## RESULTS

A total of 33 patients from seven clinical centers were evaluated, two of whom did not meet the eligibility criteria. Thirty-one patients who provided written informed consent and were treated in the Brazilian durvalumab EAP between September 2017 and June 2018 were included in this study. According to the 8th edition of the IASLC, the patients had stage IIIA to IIIC NSCLC, with adenocarcinoma being the most common histology (58.1%), followed by squamous cell carcinoma (32.3%). The median age at durvalumab initiation was 66.4 years (range 50.5 - 85.8). Baseline patient characteristics are shown in Table 1.

**Table 1:** Epidemiological and clinical features.

Characteristic	Statistics (N=31)
Median age in years (range)	66.4 (50.5-85.8)
Sex - n (%)	
Male	21 (67.7)
Female	10 (32.3)
Skin color - n (%)	
White	24 (77.4)
Black	2 (6.5)
Mixed	1 (3.2)
Unknown	4 (12.9)
Smoking status - n (%)	
Never	4 (12.9)
Former	21 (67.7)
Current	4 (12.9)
Unknown	2 (6.5)
ECOG performance status prior to chemoradiotherapy - n (%)	
0	8 (25.8)
1	17 (54.8)
2	1 (3.2)
Unknown	5 (16.1)
Tumor histological type - n (%)	
Adenocarcinoma	18 (58.1)
Squamous cell carcinoma	10 (32.3)
Adenosquamous carcinoma or NOS	3 (9.7)
Stage / AJCC Stage 8th edition - n (%)	
IIIA	10 (32.3)
IIIB	15 (48.4)
IIIC	3 (9.7)
Unknown	3 (9.7)
Primary tumor - n (%)	
T1a	3 (9.7)
T1b	1 (3.2)
T1c	1 (3.2)
T2a	3 (9.7)
T2b	2 (6.5)
T3	6 (19.4)
T4	9 (29.0)
Unknown	6 (19.4)
Regional lymph nodes - n (%)	
N0	1 (3.2)
N1	3 (9.7)
N2	13 (41.9)
N3	8 (25.8)
Nx	1 (3.2)
Unknown	5 (16.1)

Most patients were male (67.7%), former or current smokers (80.6%), and had an ECOG performance status of 0, 1, or 2 before chemoradiotherapy (25.8%, 54.8%, and 3.2%, respectively) (Table 1). All patients had comorbidities, with hypertension being the most common (11.4%), followed by diabetes (7.1%) and hypothyroidism (4.3%).

In the initial staging, 51.6% of the patients underwent CNS imaging, primarily using MRI (87.5%). Although it was not a criterion for inclusion in the study, 83.9% of the patients were evaluated with PET-CT. Invasive mediastinal staging was performed in 29% of the patients.

Among the 18 patients with adenocarcinoma, the EGFR status was available for 10 (55.6%), with 2 patients (20%) presenting EGFR mutations. The ALK status was tested in 55.6% of the participants, and the ROS status was tested in 11.1%; all of them were ALK and ROS wild-type. Unfortunately, the low rate of molecular evaluations for oncogenic mutations reflects the limited number of tests performed in our clinical practice, indicating that this practice is not yet widespread. PD-L1 expression levels were reported in 35.5% of the patients; of these, 27.2% had a PD-L1 expression <1%, and 72.3% had expression ≥1%. The predominant immunohistochemical assay used was the 22C3 pharmDx antibody kit (Dako Inc., CA, USA), in 63.6% of cases.

### Chemoradiotherapy Efficacy and Safety

Only seven patients (22.6%) received induction chemotherapy, with carboplatin combined with paclitaxel being the preferred regimen. Most patients received carboplatin-based chemoradiotherapy (80.6%). The associated agents included paclitaxel (64.5%), etoposide (19.4%), and pemetrexed (9.7%).

Overall, 20 out of the 31 patients received IMRT (64.5%), with a mean total delivered dose of 57.9 Gy. CRT interruption was not necessary for most patients (80.6%). Around 20% underwent sequential chemoradiotherapy. Adverse events (AEs) occurred in 45.2% of the patients during CRT, with the most common symptoms being dysphagia (12.9%) and nausea (12.9%). Only 1 patient (3.2%) developed pneumonitis. During definitive treatment, AEs of any grade were observed in 41.9% of the patients, with grade 3 AEs occurring in only 9.7%. Approximately 55% of these events were considered unrelated to treatment.

Most patients (83.9%) did not undergo new CNS imaging, and 64.5% did not have a new PET-CT before chemoradiotherapy. The evaluation following CRT showed that the best response was a partial response in most patients (71%). Stable disease was observed in 9.7% of the patients, with complete response in 3.2%. None of them experienced disease progression during definitive treatment. Only two patients received additional adjuvant chemotherapy after CRT (6.5%).

### Durvalumab Efficacy

**Figure 1:**
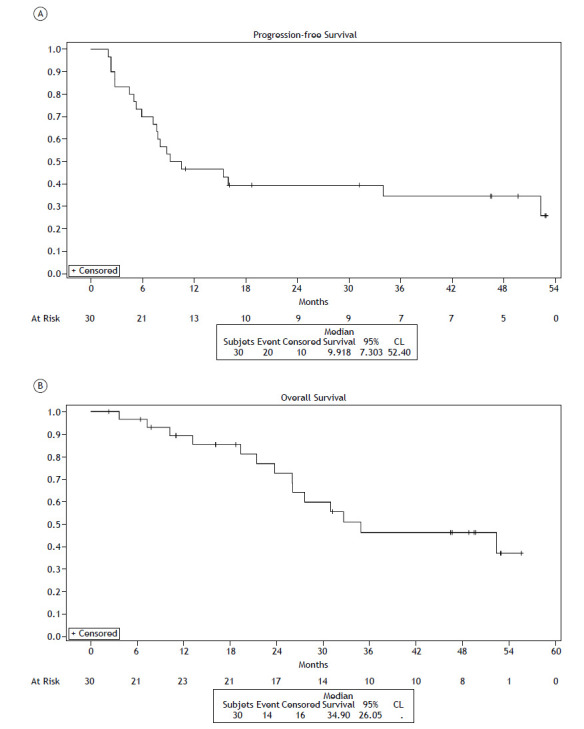
Progression-free survival (A) and overall survival (B) from the start date of durvalumab therapy. CL: confidence interval.

After a median follow-up period of 50.3 months (95% CI: 32.3 - 54.4), the median PFS from durvalumab initiation was 9.9 months (95% CI: 7.3 - 52.4) (Figure 1A). The PFS rates from the start of durvalumab at 12, 24, and 36 months were 46.7% (95% CI: 28.4 - 63.0), 39.5% (95% CI: 22.2 - 56.3), and 34.5% (95% CI: 17.7 - 52.1), respectively.

Data on survival at the third year of follow-up indicated that 51.6% of the patients had already died. Among these deaths, 75% were related to cancer progression. The median OS was 34.9 months (95% CI: 26.0 - NR) in the overall population (Figure 1B). The OS rates from the start of durvalumab at 12, 24, and 36 months were 89.3% (95% CI: 70.3 - 96.4), 72.6% (95% CI: 50.7 - 86.0), and 46.3% (95% CI: 25.7 - 64.6), respectively.

**Figure 2:**
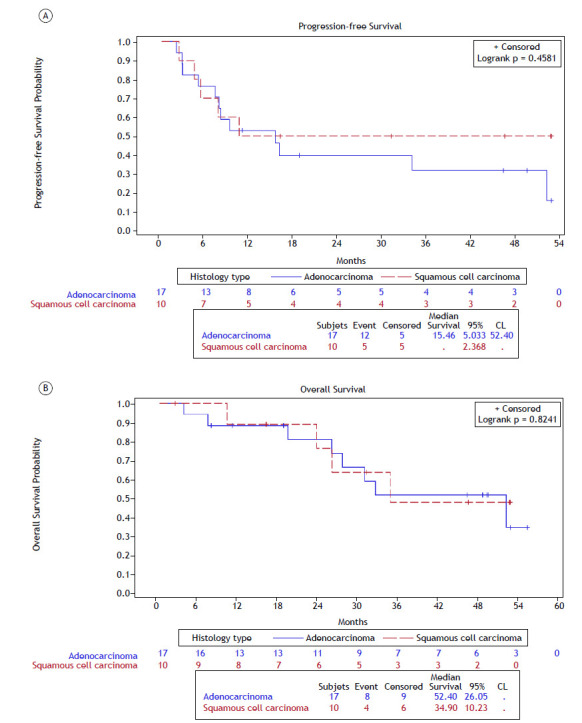
Progression-free survival (A) and overall survival (B) by histological subtype from the start date of durvalumab therapy. CL: confidence interval.

Patients with adenocarcinoma had a longer median OS than those with squamous cell carcinoma, although this difference was not statistically significant. The median PFS was 15.4 months (95% CI: 5.0 - 52.4) for adenocarcinoma, while it was not reached (95% CI: 2.3 - NR) for squamous cell carcinoma (Figure 2A). The median OS was 52.4 months (95% CI: 26.0 - NR) for adenocarcinoma and 34.9 months (95% CI: 10.2 - NR) for squamous cell carcinoma (Figure 2B). This study lacked the statistical power to detect differences in survival between the different histologies due to the small sample size. The patients were evaluated during consolidation therapy, with the best responses as follows: 9.7% showed a complete response, 19.4% showed a partial response, 19.4% had stable disease, and 19.4% had progressive disease. The best response could not be assessed in approximately 32% of the patients.

### Durvalumab Compliance and Safety

The median duration of treatment with durvalumab was 9.7 months (IQR: 6.0 - 11.1), with a median of 17 cycles (10 to 24). The median time to start immunotherapy after chemoradiation was 1.86 months (IQR: 1.18 - 2.89). Temporary interruption was necessary in 16.1% of cases, with the main causes being toxicity and suspected active infection. Approximately half (45.2%) of the patients completed 12 months of planned durvalumab therapy. Among the 17 patients who were unable to complete definitive treatment, 15 (88.2%) had progressive disease, 1 (5.9%) lost follow-up, and 1 (5.9%) died from complications of a second primary tongue cancer.

During consolidation immunotherapy, 61.3% and 38.7% of the patients reported experiencing AEs and treatment-related AEs, respectively. The most common AEs related to durvalumab were pain and cough (16.1%), followed by pneumonitis and AST/ALT abnormalities (12.9%), hypothyroidism, dyslipidemia, fatigue, pruritus, and localized urticaria (6.4%), and anemia, myalgia, pulmonary thrombosis, hypotension, nausea, decreased visual acuity, inappetence, upper airway obstruction, and vomiting (3.2%). Most AEs were grade 1 or 2 (97.3%), with only 1 (2.7%) being grade 3. Those related to immunotherapy are shown in Table 2.

**Table 2:** Treatment-related adverse events during consolidation therapy.

Adverse event	Total n (%)	Grade 1 n (%)	Grade 2 n (%)	Grade 3 n (%)
Cough	5 (16.1)	4 (12.9)	1 (3.2)	0 (0.0)
Pain	5 (16.1)	5 (16.1)	0 (0.0)	0 (0.0)
Pneumonitis	4 (12.9)	1 (3.2)	2 (6.4)	1 (3.2)
AST/ALT abnormality	4 (12.9)	4 (12.9)	0 (0.0)	0 (0.0)
Hypothyroidism	2 (6.4)	1 (3.2)	1 (3.2)	0 (0.0)
Dyslipidemia	2 (6.4)	2 (6.4)	0 (0.0)	0 (0.0)
Fatigue	2 (6.4)	2 (6.4)	0 (0.0)	0 (0.0)
Pruritus	2 (6.4)	2 (6.4)	0 (0.0)	0 (0.0)
Localized urticaria	2 (6.4)	1 (3.2)	1 (3.2)	0 (0.0)
Anemia	1 (3.2)	0 (0.0)	1 (3.2)	0 (0.0)
Myalgia	1 (3.2)	1 (3.2)	0 (0.0)	0 (0.0)
Pulmonary thrombosis	1 (3.2)	0 (0.0)	1 (3.2)	0 (0.0)
Hypotension	1 (3.2)	1 (3.2)	0 (0.0)	0 (0.0)
Nausea	1 (3.2)	1 (3.2)	0 (0.0)	0 (0.0)
Reduced visual acuity	1 (3.2)	1 (3.2)	0 (0.0)	0 (0.0)
Inappetence	1 (3.2)	1 (3.2)	0 (0.0)	0 (0.0)
Upper airway obstruction	1 (3.2)	1 (3.2)	0 (0.0)	0 (0.0)
Vomiting	1 (3.2)	1 (3.2)	0 (0.0)	0 (0.0)

The percentages presented in this Table were calculated using the total number of patients (N=31) as the denominator.

Regarding the nine immune-mediated AEs observed during consolidation therapy, there were 4 reported cases of pneumonitis (12.9%), 3 of hypothyroidism (9.7%), and 1 of pruritus (Table 3). Systemic steroids were used in 5 patients, and no other immunosuppressive agent was required. The frequency of pneumonitis was similar to that reported in other studies involving immune checkpoint inhibitors. One patient had grade 3 pneumonitis, and no cases of grade 4 or 5 were reported.

**Table 3:** Immune-mediated adverse events during consolidation therapy.

Adverse event	Total n (%)	Grade 1 n (%)	Grade 2 n (%)	Grade 3 n (%)
Pneumonitis	4 (12.9)	1 (3.2)	2 (6.4)	1 (3.2)
Hypothyroidism	3 (9.7)	1 (3.2)	2 (6.4)	0 (0.0)
Pruritus	1 (3.2)	1 (3.2)	0 (0.0)	0 (0.0)

The percentages presented in this Table were calculated using the total number of patients (N=31) as the denominator.

Among the patients whose disease progressed, the most affected sites were distant lymph nodes (27.8%), brain (11.1%), bone (11.1%), adrenal glands (11.1%), lungs (5.6%), mediastinum (5.6%), and pleura/pericardium (5.6%). The frequency of CNS implants during the study period was 12.9%. Among these patients, half experienced neurological symptoms related to their disease.

Approximately 70% of the 31 patients with disease progression underwent subsequent systemic therapy. Among these 12 patients, a total of 41.7% received at least one line of treatment, 33.3% received two lines, 16.7% received three lines, and 8.3% received four lines. Chemotherapy was the primary choice of systemic treatment (74.9%), while 16.7% of patients received Osimertinib, and 8.3% received Crizotinib. Additionally, 16.1% of the patients received palliative radiotherapy.

## DISCUSSION

To the best of our knowledge, LACOG 0120 is the first study to provide real-world data on Brazilian patients who received durvalumab through an EAP. Conducted across seven Brazilian centers, this study analyzed the treatment outcomes of patients treated with the PACIFIC regimen. The median PFS was 9.9 months, lower than the results reported in the European (PACIFIC-R)^10^ ^)^ and Korean (PACIFIC-KR)^13^ studies, with median PFS values of 21.7 and 25.9 months, respectively. The median PFS observed herein was also lower than the 5-year analysis of the PACIFIC trial, at 9.9 months compared to 16.9 months.^7^

The mean age of the patients in our study was approximately 65 years, similar to findings reported in other studies.^10^ ^,^ ^13^ Former and current smokers accounted for roughly 80% of the patients, comparable to Western and Korean data, where smokers represented approximately 90% and 80%, respectively.^10^ ^,^ ^13^ Adenocarcinoma was the predominant histology, except in the Korean population, which reported 52.2% of cases as squamous cell carcinoma.^14^ Among the patients in LACOG 0120, 58.1% had stage IIIB or IIIC disease, reflecting a cohort with more advanced disease compared to the PACIFIC-R study.^10^ In contrast, a German study included patients with stage IV disease.^11^ In the present study, initial evaluations showed a higher usage of PET-CT and MRI, suggesting a growing preference for advanced imaging modalities during the initial staging and assessment of these patients.

The European population had a 2-year OS rate of 71.2%,^10^ while the Korean population reported 71%.^14^ In the PACIFIC study population, the 2-year OS rate was 63.3%, lower than the rates observed in real-world data analyses, including the Brazilian cohort, which had a 2-year OS rate of 72.6%.^7^ ^,^ ^10^ ^-^ ^13^

In real-world studies, high expression of PD-L1 was associated with better survival rates, and patients with mutated EGFR showed less benefit from consolidation ICI after CRT.^9^ ^-^ ^13^ Our study had similar rates of EGFR mutations and PD-L1 expression. Despite these similarities, the trial lacked the statistical power to detect differences between subgroups due to the small number of patients.

In these populations, chemotherapy regimens were mostly based on platinum, with a predominance of carboplatin use in the Brazilian population, which differed from the European and Korean populations, where cisplatin was predominantly used. There were no major differences in response rates or toxicity related to these variables.^10^ ^-^ ^14^ The number of patients who completed the durvalumab consolidation regimen was similar across the samples. The European population received a higher number of durvalumab doses compared to the Brazilian study (22 vs. 17.9 doses).^10^ In spite of the higher dose density in the PACIFIC-R population and its higher PFS, the 2-year survival rates were very similar when compared to LACOG 0120.^10^ ^)^ Some important limitations worth mentioning in evaluating our results include the limited sample size and the low rate of molecular assessments to evaluate somatic driver mutations.

Adverse events occurred in 61.3% of the patients in our study, with most being grade 1 or 2. Regarding immunotherapy-related pneumonitis, 4 cases (12.9%) were reported, with only 1 case classified as grade 3 (3.2%). Our study did not reveal any new safety signals, and when compared to the PACIFIC trial, it showed a lower rate of adverse events, including pneumonitis, and a lower rate of durvalumab discontinuation.^7^ ^,^ ^8^

Our study reported no new safety signals regarding immune-related AEs, even in the context of low-middle-income countries with limited resources for diagnosing and managing such events. In addition, our patients were treated within the public health system, which faces challenges in patient awareness and access, particularly regarding treatment-related complications. Systemic steroids were used in 16% of the patients, and no need for any other immunosuppressive agent was noted.

## Data Availability

The study protocol and data, including trial-level data (analysis datasets), as well as other information (*e.g*., clinical study reports or analysis plans), are available upon request from the investigators. These clinical trial data can be requested by any qualified researcher engaged in rigorous, independent scientific research and will be provided following the review and approval of a research proposal, statistical analysis plan, and execution of a data-sharing agreement. Data requests can be submitted to the corresponding author at any time after the acceptance of this manuscript for publication.
